# Conservative treatment in Scheuermann’s kyphosis: comparison between lateral curve and variation of the vertebral geometry

**DOI:** 10.1186/s13013-016-0089-4

**Published:** 2016-10-14

**Authors:** Angelo G. Aulisa, Francesco Falciglia, Marco Giordano, Giuseppe Mastantuoni, Andrea Poscia, Vincenzo Guzzanti

**Affiliations:** 1 U.O.C. of Orthopedics and Traumatology, Children’s Hospital Bambino Gesù, Institute of Scientific Research, Rome, 00165 Italy; 2 School of Hygiene and Preventive Medicine, University Hospital “Agostino Gemelli”, Catholic University of the Sacred Heart School of Medicine, Rome, 00168 Italy; 3 University of Cassino, Cassino, (FR) 03043 Italy

## Abstract

**Background:**

Conservative treatment in the Scheuermann’s kyphosis obtain, during skeletal growth, remodelling of the deformed vertebras. In a previous paper on Scheuermann’s kyphosis, we have studied the geometry variations of all vertebrae included in the curve, before and after the treatment.

The purpose of this study was to confirm the effectiveness of conservative treatment in Scheuermann’s kyphosis and was to evaluate and compare the variation of the vertebral geometry with the curve trend in Cobb degrees, before and after conservative treatment.

**Methods:**

From a consecutive series of patients, we selected 90 patients with thoracic Scheuermann’s kyphosis, treated using anti-gravity brace: 59 male, 31 female. The mean age at the beginning of the treatment was 14 years.

Radiographical measurements were performed on radiographs from a lateral projection, at the beginning (t1) and at the end of the treatment (t5). Vertebral geometry modifications at t1 and t5 were analysed according to the following parameters and evaluated by three independent observers: Anterior wedging angle (ALFA) of the apex vertebra and Posterior wall inclination (APOS) of the limiting lower vertebra. The curve was measured in Cobb degrees.

**Results:**

The results from our study showed that of the 90 patients with a thoracic curve mean value of Cobb degrees was 57.8 ± 6.0 SD at t1 and 41.3 ± 5.6 SD at t5. The differences between t1(angle at baseline) and t5 (end of treatment) were calculated for Cobb, ALFA and APOS angle and were respectively −16.4 ± 4.5, −6.4 ± 1.4 and −2.7 ± 1.2; tested with paired *t*-test were significative (*p* < 0.01). The results of the regression analysis to test the relationship between the three measures for the kyphosis (Cobb degree, ALFA and APOS) showed that the best association was between Cobb t5 and ALFA t5 (*p* < 0.01) and Cobb t1 and APOS t1 (*p* < 0.01). No significative association was found between the difference between ALFA and APOS.

**Conclusion:**

We sustain that using new parameters to study vertebral remodelling allows us to reach a better comprehension of Scheuermann spine response to anti-gravity brace treatment. Furthermore, the evaluation of the ALFA angle of the apex vertebra confirms to be more reliable than Cobb’s angle because it cannot be affected by the radiological position.

## Background

In 1920 Scheuermann [1] first described the association of developmental Kyphosis and wedging of thoracic vertebrae; he used the term “osteochondritis juvenilis dorsi” [2], but the condition is universally known today as Sheuermann’s kyphosis. Sorenson [3], proposed a diagnosis based on the presence of three or more adjacent vertebrae wedged 5° or more and no evidence of congenital, infectious or traumatic disorders of the spine. These criteria are widely accepted and used today. The prevalence in the general population ranged from 4 to 10 % [4, 5]. The pathogenesis is still not clear some authors write that “The weakness of the vertebral endplate probably results from a predisposing genetic background that influences the quality of matrix components (collagen types II and IX) and chondrocytes” [5, 6] other said that mechanical stress influences the severity of spinal impairment [7].

Vertebral geometry alterations in Scheuermann’s kyphosis and results of the orthopedic treatment have been measured by radiographic measure of both curve entity and vertebral wedging on longitudinal section [8–11]. Clinical evolution of the deformity is not always correlated to presently used radiographic parameters. On the other hand, it is possible that vertebal morphology alteration in kyphotic curve could be explained by a more complex theory model than the currently accepted one [12]. For this reason, in a previous paper on Scheuermann’s kyphosis, we have studied the geometry variations of all vertebrae included in the curve, before and after the treatment [13].

The purpose of this study was to confirm the effectiveness of conservative treatment in Scheuermann’s kyphosis and was to evaluate and compare the variation of the vertebral geometry with the curve trend in Cobb degrees, before and after conservative treatment.

## Methods

**Fig. 1:**
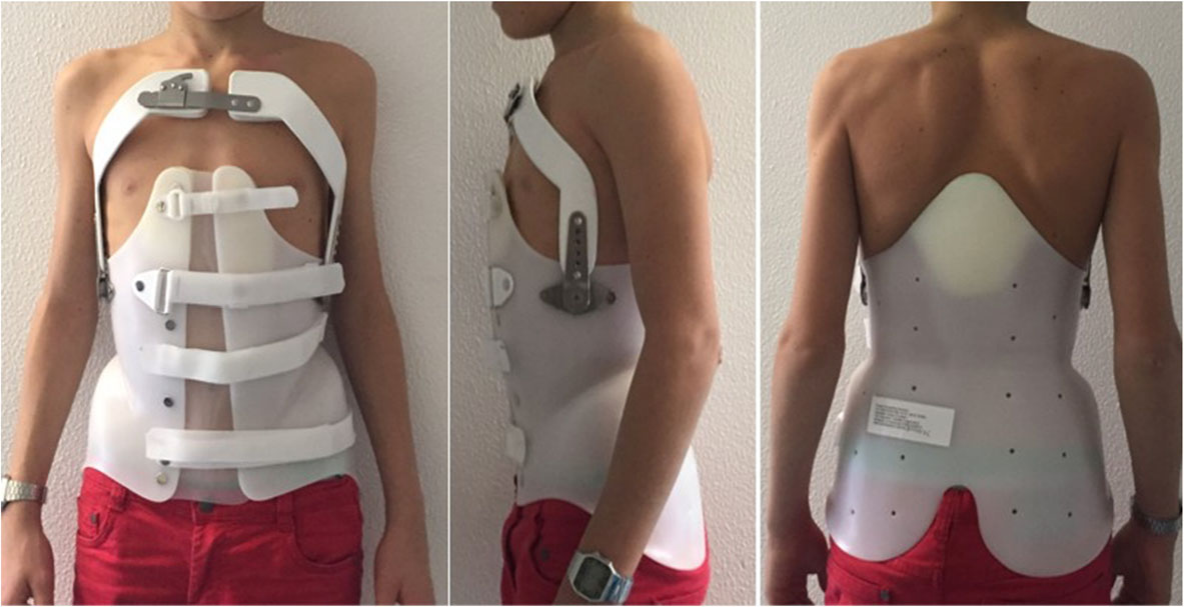
The anti-gravity brace used for the treatment of Scheuermann’s kyphosis

We selected, from a consecutive series of patients, included in a prospective database, 90 patients with thoracic Scheuermann’s kyphosis, treated using anti-gravity brace between 2004 and 2010 (Fig. 1). Other type of kyphosis were excluded. 59 patients were male, 31 were female. The mean age at the beginning of the treatment was 14.2 ± 1.8 years. The mean curve entity before treatment, measured by Cobb’s method, was 57.8 °, a value that, according to the literature data, requires orthopaedic treatment [14].

All cases were treated with anti-gravity brace. Time bracing prescribed was max 20 h daily, min 16 h daily. In order to maximize the adherence to treatment, patients were always followed by the same doctor. Furthermore, controls were performed every 3 months. Frequent checks allowed to verify and implement compliance establishing an open and friendly relationship with the patients. Close checks were also performed to maximize bracing effectiveness over the time. Weaning was started when a full recovery of vertebral geometry was seen on a latero-lateral radiograph view. Not exercises were performed.

Radiographical measurements were performed on radiographs from a lateral projection, at the beginning (t1) and at the end of the treatment (t5).

To avoid the great variance in the range of curve angles in thoracic kyphosis that rely on the radiological position, x-rays were performed all at our Radiology Department observing the following position: standing with head straight, arms bent at 45° and hands placed on a support.

Vertebral geometry modifications at t1 and t5 were analysed according to the following parameters and evaluated by three independent observers:

- Cobb degrees for curve magnitude
- Anterior wedging angle (ALFA) of the apex vertebra
- Posterior wall inclination (APOS) of the limiting lower vertebra.

**Fig. 2:**
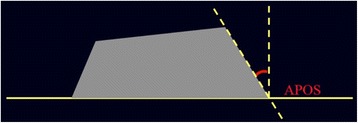
Measure of posterior wall inclination APOS, conducted using disk plate limits of every vertebra. More specifically, we have calculate the angles between the line perpendicular to inferior plate and the line passing trough superior and inferior limit of posterior wall

In particular, the measurement of ALFA angle was the calculation of the convex angle formed by two lines perpendicular to the lines passing through posterior and anterior limit, respectively of superior and inferior disk plates of the vertebral body (Fig. 2). Instead the measurement of the posterior wall inclination APOS, was conducted using disk plate limits of each vertebra. More specifically, we have calculated the angles between the line perpendicular to the inferior plate and the line passing through superior and inferior limit of posterior wall.

These parameters were chosen because they had shown to be the most significant in a previous study (13).

### Statistical analysis

Mean and Standard Deviation of kyphosis degree as Cobb, ALFA and APOS angle at baseline (t1) and at the end of treatment (t5) were calculated and differences between t1 and t5 were tested with paired *t*-test. Furthermore, a linear regression model was used to test the relationship between Cobb, ALFA and APOS angle at t1, t5 and at t5-t1 difference.

## Results

**Fig. 3:**
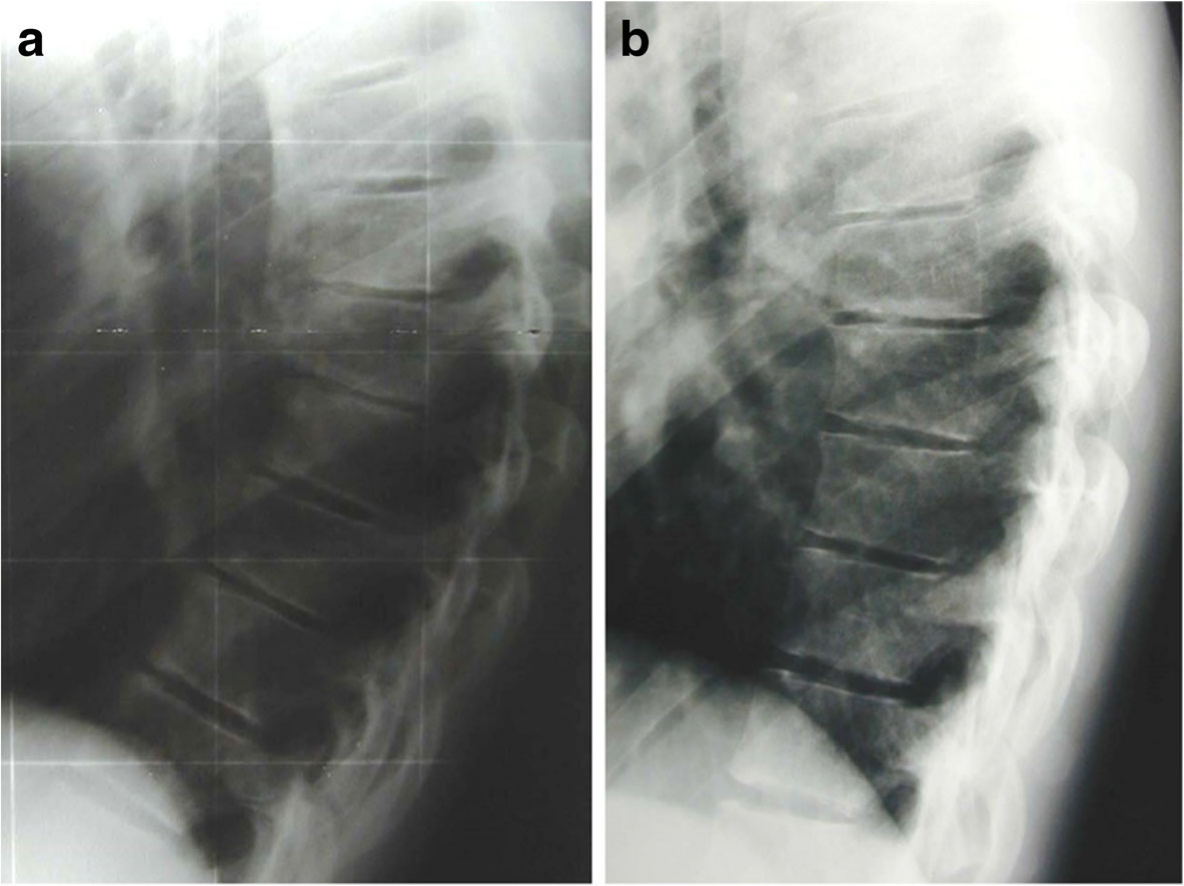
The figure shows a case at beginning of treatment (**a**) and at follow-up (**b**)

The results from our study showed that of the 90 patients with a thoracic curve mean value of Cobb degrees was 57.8 ± 6.0 SD at t1 and 41.3 ± 5.6 SD at t5 (Fig. 3). The mean duration of treatment was 32.9 ± 18.44 months and the mean follow-up was 30.02 ± 18.85 months. The differences between t1(angle at baseline) and t5 (end of treatment) were calculated for Cobb, ALFA and APOS angles and were respectively −16.4 ± 4.5, −6.4 ± 1.4 and −2.7 ± 1.2; tested with paired *t*-test were significative (*p* < 0.01) (Table 1). No difference statistically significative between male and female was reported. The results of the regression analysis to test the relationship between the three measures for the kyphosis (Cobb degree, ALFA and APOS) are shown in Table 2. The best association was found between Cobb t5 and ALFA t5 (*p* < 0.01) and between Cobb t1 and APOS t1 (*p* < 0.01). No significative association was found between the difference between ALFA and APOS.

**Table 1:** Mean, Standard Deviation (SD) of kyphosis in terms of Cobb degree, ALFA and APOS angle at baseline (t1) and at the end of treatment (t5)

*Condition*	*Time*	*Mean*	*sd*	*Differ ence T5-T1 mean (SD)*	*P value (Paired T test)*
*Cobb*	t1	57.8	6.0	−16.4 (4.5)	<0.01
	t5	41.3	5.6		
*ALFA ant*	t1	14.0	2.2	−6.4 (1.4)	<0.01
	t5	7.6	2.4		
*APOS*	t1	4.6	1.7	−2.7 (1.2)	<0.01
	t5	1.9	1.0

**Table 2:** Regression analysis to test the relationship between Cobb, ALFA and APOS angle

*Y*	*X*	*Coeff*	*p*	*R2*
*Cobb t1*	ALFA t1	1.63	<0.01	0.3545
*Cobb t5*	ALFA t5	2.03	<0.01	0.7610
*Cobb t5-t1*	ALFA t5-t1	0.81	0.019	0.0605
*Cobb t1*	APOS t1	2.72	<0.01	0.6160
*Cobb t5*	APOS t5	2.89	<0.01	0.2762
*Cobb t5-t1*	APOS t5-t1	1.38	<0.01	0.1322
*ALFA t5-t1*	APOS t5-t1	−0.20	0.09	0.0307

## Discussion

The results confirm that conservative treatment in Scheuermann’s Kyphosis, during skeletal growing, is effectiveness and we can obtain a remodelling of the deformed vertebrae.

In particular the antigravity brace, based on biomechanical action of the three points principle: one force is applied behind the curve apex and the other two forces are applied at the end of the vertebrae at the curve ends. Moreover, in according to the vector calculation principles, a force applied to a curve structure is divided in two components with direction and course determined by the application point and by the space orientation of the resultant. Therefore it is logical that forces applied by an antigravity brace could produce different effects on the vertebral remodelling, depending on the vertebral position. On this basis, we sustain that using ALFA angle of the apex vertebra and APOS angle of the limiting lower vertebra to study vertebral remodelling allows us to reach a better comprehension of Scheuermann spine response to anti-gravity brace treatment.

## Conclusion

The correlation between Cobb and ALFA at follow-up and Cobb and Posterior Wall Inclination at baseline confirm the complexity of vertebral remodelling and allows us to reach a better comprehension of Scheuermann spine response to anti-gravity brace treatment.

Moreover the evaluation of the ALFA angle of the apex vertebra confirms to be more reliable than Cobb’s angle because it cannot be affected by the radiological position.

## Abbreviations

ALFA: Anterior wedging angle
APOS: Posterior wall inclination
